# Surgery goes EPA (Entrustable Professional Activity) – how a strikingly easy to use app revolutionizes assessments of clinical skills in surgical training

**DOI:** 10.1186/s12909-022-03622-1

**Published:** 2022-07-19

**Authors:** Nadine Diwersi, Jörn-Markus Gass, Henning Fischer, Jürg Metzger, Matthias Knobe, Adrian Philipp Marty

**Affiliations:** 1 Department of General Surgery, Cantonal Hospital of Lucerne, Spitalstrasse 16, 6000 Lucerne, Switzerland; 2 Department of Health Sciences and Medicine, University of Lucerne, 6002 Lucerne, Switzerland; 3 Institute of Anesthesiology, University Hospital Zürich, Rämistrasse 100, 8006 Zürich, Switzerland; 4 precisionED Ltd, Muehlebachstrasse 2, 8832 Wollerau, Switzerland

**Keywords:** Mobile application, Performance assessment, Surgical training, Entrustable professional activity

## Abstract

**Objective:**

Entrustable Professional Activities (EPAs) are increasingly being used in competency-based medical education approaches. A general lack of time in clinical settings, however, prevents supervisors from providing their trainees with adequate feedback. With a willingness for more administrative tasks being low in both trainees and educators, the authors developed a radical user-friendly mobile application based on the EPA concept called “Surg-prEPAred”.

**Design:**

Surg-prEPAred is designed to collect micro-assessment data for building competency profiles for surgical residents according to their curriculum. The goal of Surg-prEPAred is to facilitate the performance and documentation of workplace-based assessments. Through aggregated data the app generates a personalized competency profile for every trainee. During a pilot run of 4 months, followed by ongoing usage of the application with a total duration of 9 months (August 2019 to April 2020), 32 residents and 33 consultants made daily use of the application as a rating tool. Every rating included knowledge, skills and professional attitudes of the trainees. Before the initiation of the App and after the 9-month trial period trainees and supervisors where both sent questionnaires to evaluate the user friendliness and effectiveness of the App.

**Results:**

Five hundred ten App based assessments were generated. Out of 40 pre-defined EPAs, 36 were assessed. 15 trainees and 16 supervisors returned the questionnaires and stated the surg-prEPAred App as very valuable, effective and feasible to evaluate trainees in a clinical setting providing residents with an individual competence portfolio to receive precision medical education.

**Conclusions:**

The authors expectation is that the Surg-prEPAred App will contribute to an improvement of quality of medical education and thus to the quality of patient care and safety. In the future the goal is to have the App become an integral part of the official Swiss surgical curriculum accepted by the Swiss professional surgical society.

**Supplementary Information:**

The online version contains supplementary material available at 10.1186/s12909-022-03622-1.

## Background

 “Entrustable Professional Activities” (EPAs) were initially introduced in 2005 and are increasingly being used in competency-based medical education approaches [1, 2]. An EPA is defined as a unit of professional practice that can be fully entrusted to a trainee, as soon as he or she has proven the required capability to perform the activity without supervision [3]. Depending on the field of specialty chosen, a set of EPAs necessary to master can be pre-defined by medical educators, each EPA consisting of a set of competencies needed for the task at hand. The task of monitoring and documenting the learning progress of each trainee has been proven to be a challenge [4–8]. In a more and more demanding clinical setting with a general lack of time and thus motivation to evaluate trainees, an easy-to-use tool is required to facilitate a more efficient and meaningful feedback culture among trainees and educators. We believe that mobile applications increase feedback numbers, support entrustment decision-making and also visualizes a trainee’s level of expertise more adequately. To our knowledge, only two references on combining the EPA framework with a mobile platform can be retrieved in literature [9, 10]. Whereas the published paper focuses on second year psychiatry residents, we would like to discuss our mobile app Surg-prEPAred which we designed for our surgical resident curriculum at the Kantonsspital Luzern, the largest non-university hospital in Switzerland. The following paper describes both implementation and results after a pilot run of 4 months, followed by ongoing usage of the application with a total duration of 9 months (August 2019 to April 2020).

### Intention and background information

A lot of alternative forms of workplace-based assessments such as “Direct Observation of Procedural Skills” (DOPS) and “Mini-Clinical Evaluation Exercise” (Mini-CEX) are well-founded from a theoretical teaching perspective, but they are difficult to implement within the clinical setting [11]. They are said to be too much of a checklist approach to medical education, while leaving out important competencies with relevant differences that are difficult to measure [6, 12–15]. Numerous studies have shown that assessments and feedback are rarely done on a regular basis and often show a lack of advice on how the trainee can improve her/his clinical expertise [12, 16]. Very often the competencies of a trainee are also fragmented to subgroups, such as communication skills, knowledge, manual skills, etc. without focusing on the greater picture. Thus, the concept of Entrustable Professional Activities is better suited for everyday clinical life. The focus of EPAs lies on the holistic rating of a specific and observable clinical task [1]. The level of competency is the level of required supervision, a scale that educators use implicitly every day, but failed to document till now. However, each clinical task is suitable for learning and its assessment should be used as a teaching tool with only little effort. With the understanding that the willingness for more administrative tasks is very low in both trainees and educators, we developed a radical user-friendly mobile application based on the EPA concept called “Surg-prEPAred”. The app has been developed using a design-thinking approach. To ensure user-centered design, the original concept by one of the authors (APM) has been improved through prototyping, testing and refining in several cycles including all stakeholders. With grant money from the University of Zurich’s “Competitive Teaching Grant” and a grant from the Swiss Institute for Postgraduate and Further Education in Medicine (SIWF), a first functional prototype was developed by an external software company in 2019.

In fall 2020, APM founded a company (precisionED Ltd) to rebuild the App from scratch and to provide a sustainable high quality assessment system. precisionED holds all intellectual property rights and guarantees state-of-the art protection of any data by complying with GDPR-standards.

Starting in July 2019, we motivated all surgical staff, trainees and consultants as educators, of the Cantonal Hospital of Lucerne to download and use the App on pre-defined EPAs during their daily business. Taking part in the pilot study was not mandatory. The only technical tool necessary was a smartphone. Each rating was set to take less than 2 minutes per case. In alignment of the EPA concept every rating included knowledge, skills and professional attitudes of the trainees. Trainee and educator were able to use the App together during their clinical work. A trainee would select the task and show a generated QR code on her/his smartphone for the educator to scan with the supervisor version of the App. After an independent rating of the trainee’s performance by both trainee and educator, specific feedback and documentation of a learning goal were an optional part of the rating process facilitated by the prEPAred-App.

In order to better assess possible improvements, a survey on the status quo of the feedback quality was sent to all users prior to the pilot study and at the end. The pre- and post-feedback surveys, as well as the usability surveys are derived from the feedback literature and common usability questionnairs [17]. They were pilot-tested by the authors, trainees and supervisors for clarity and feasibility (Questionnaires are provided in the Additional file 1). By accumulating the collected workplace-based assessment data during the 4-month pilot project phase, each trainee generated an individual, color-coded competency profile.

**Fig. 1:**
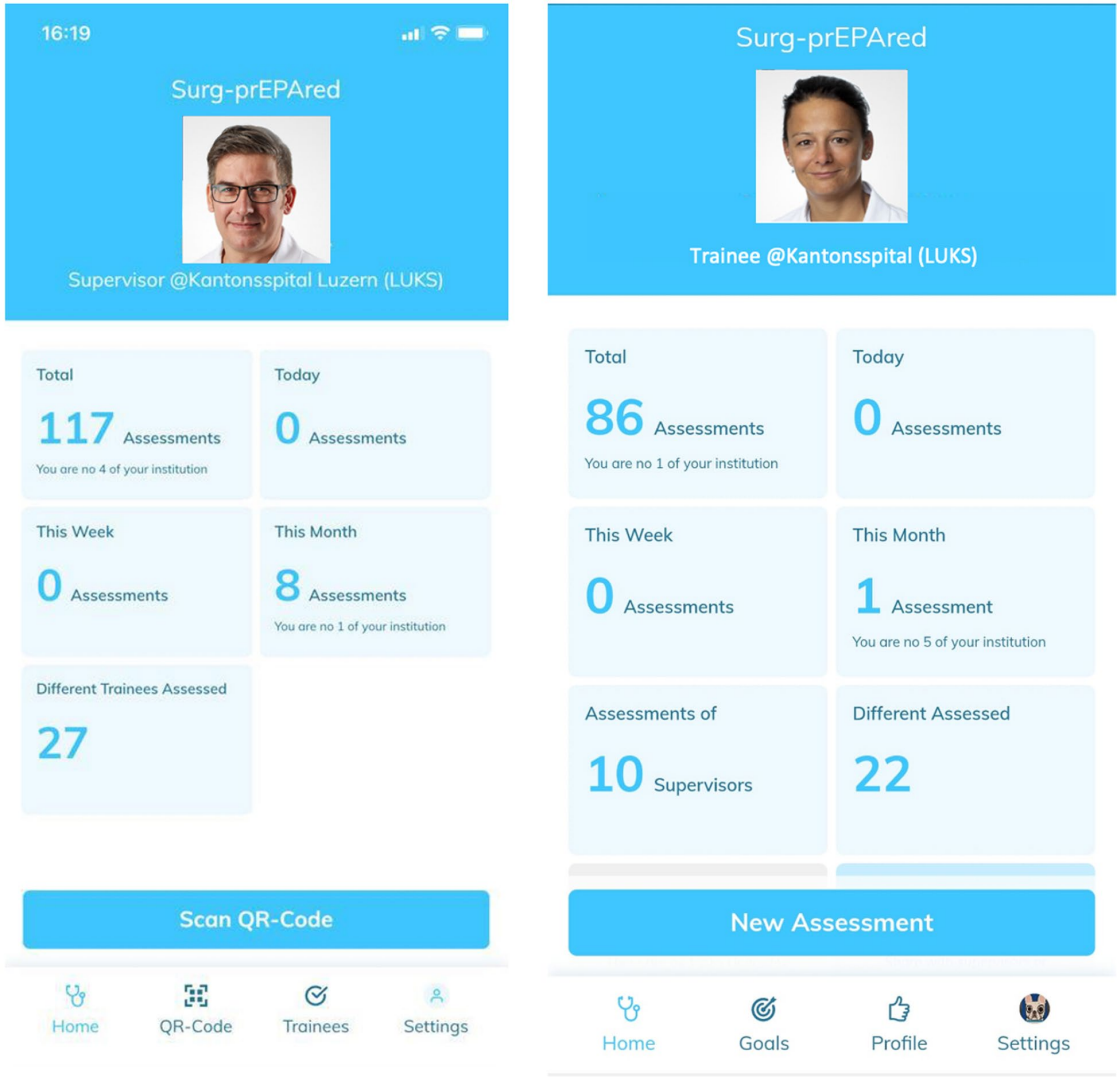
User interface of supervisor (left) and trainee (right)

**Fig. 2:**
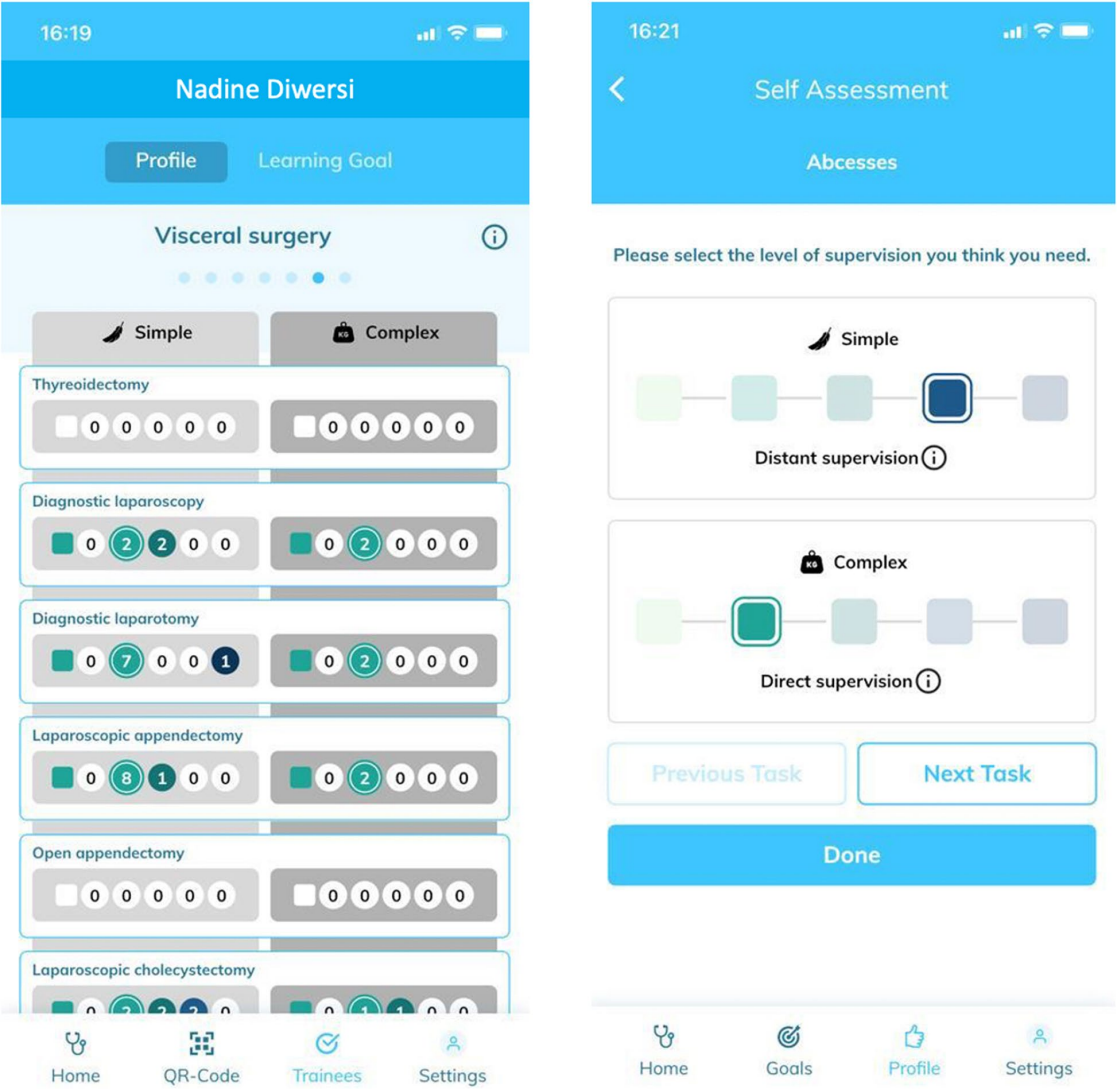
Example of a trainee competency profile and self-assessment in visceral surgery

Figure 1 shows the intuitive user interface for both supervisors on the left and trainees on the right, whereas Fig. 2 illustrates an example for a trainee competency profile on the left and the self-assessment tool on the right.

## Aims of the new assessment tool

The main goal of the Surg-prEPAred-App was to facilitate the performance and documentation of workplace-based assessments of short clinical tasks (EPAs). Through the aggregated data the app generates a personalized competency profile for every trainee.

This transparency is supposed to help to identify gaps and strengths of a trainee and allows supervisors in the busy clinical setting to tailor their supervision and teaching more efficiently to the needs of the trainee. Surg-prEPAred-App is feasible and effective assessment tool that can replace time consuming and less competency-based former assessment tools. The automatically generated competency profile belongs to the trainee and shows her/his individual strengths and performance gaps as well as significant short-term learning objectives. In addition, the App offers each trainee the opportunity for self-assessment, an essential and important feedback mechanism [18]. Each trainee has maximum control over her/his competency profile. The trainee can allow the educator to have access to the personal competency profile and also take it along when rotating through different sub-specialties or when changing teaching institutions. In this way learning outcomes can be individually adjusted to a person’s needs and redundancies can be avoided. With access to a trainee’s personal competency profile, an educator can customize educational goals and adjust levels of supervision depending on the level of competency from direct supervision to distant or no supervision. We expect that this leads to an improvement of educators` motivation as well as, better use of resources and even higher safety for patients.

## Implementation and results

In the beginning the application was introduced at the department of orthopedics and traumatology as well as the department of general and visceral surgery of the Cantonal Hospital of Lucerne. 50 of the participating residents were registered as trainees, 23 of which were inexperienced (0–2 years of postgraduate training), 17 intermediate (2–4 years of training) and 10 experienced (more than 4 years of clinical experience). Out of the 40 consultants and senior consultants as educators 1 was considered an inexperienced supervisor (less than 1 year after completing resindency), 18 intermediate (few years of experience) and 21 experienced (supervisor for many years). During the trial period between August 2019 and April 2020 a total of 510 App based assessments were generated, which is about 60 per month. Out of the pre-defined 40 relevant EPAs, 36 were assessed.

In comparison there were significantly less paper-based competency-based assessments done in the same number of months before the launch of the App. Since not all institutions keep statistics on what paper-based assessments were done, the exact number is hard to tell. Generally, there are an estimated 150 paper-based assessments done over the same amount of time. It is very likely that the minimum number of assessments required per year – in Switzerland 4 - is not met by most institutions. According to international consensus meetings, it is beneficial to have short assessments more frequent, instead of longer assessments at irregular intervals [17]. Thus, the Surg-prEPAred App is an easily applicable and efficient tool for assessing trainees in the actual clinical setting. There has been an increase in the number of assessments by about 340%.

### Analysis of user-friendliness through pre- and post-trial questionnaires

Before the initiation of the App and after the 9-month trial period trainees and supervisors where both sent questionnaires to evaluate the user friendliness and effectiveness of the App. A total number of 15 trainees and 16 supervisors returned the questionnaires with the following results:

Trainees (*n* = 15):

- 93% want to use the App often
- 93% say, the App is easy to use
- 100% claim they learned quickly how to use it
- 50% think assessments by App are better than the old paper-based assessments (50% being neutral towards both)
- 93% think the usability of the App is good to excellent

Out of those trainees that did not use the App, 29% claimed to have been too busy otherwise, 12% had second thoughts about data security, 29% were on an external rotation, and 18% claimed to not have the technical equipment to do so.

The free text fields mainly proved that excitement and approval for the App were dominant attitudes, that more detailed feedback is important and that a culture change for feedback conversations is imminent.

Supervisors (*n* = 16):

- 93% want to use the App often
- 93% say, the App is easy to use
- 87% claim they learned quickly how to use it
- 66% think assessments by App are better than the old paper-based assessments (27% being neutral towards both)
- 93% think the usability of the App is good to excellent

Out of those supervisors that did not use the App, one person claimed to have been too busy to do so, another said no trainee asked to be assessed and a third person is a supervisor in the orthopedic department that has not yet been integrated into the project.

The free text fields showed constructive feedback such as that feedback should be made obligatory for each EPA assessment and some supervisors showed second thoughts concerning the low complexity of the assessments and wondered if they are detailed enough to decide about competence levels and necessary level of supervision.

### Effect on feedback quality

Pre- and post-trial questionnaires showed an increase in feedback given in addition to the assessment by 20% with immediate feedback being given 20% more often than before. The specificity of the feedback increased by 11 and 31% agreed that the feedback includes an action plan of how to improve in the future. It is usually the supervisors that dominate the feedback rounds. The quality of the feedback increased by 24% towards high quality feedback. 17% of the trainees were more satisfied with the feedback given at the end of the 9-month trial period than before usage of the App.

### Other key figures evaluated

#### Duration of rating

**Fig. 3:**
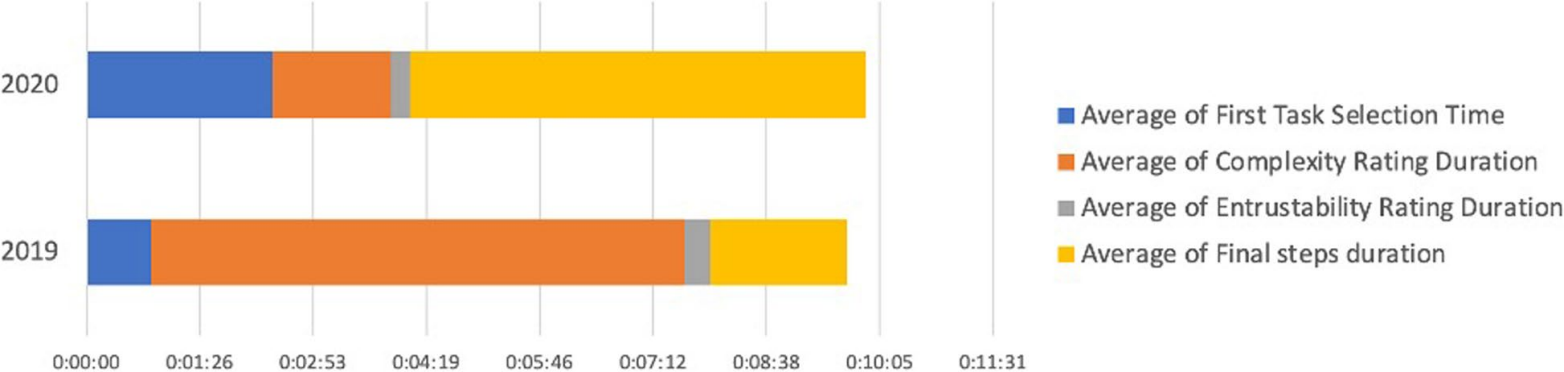
Average duration of ratings for trainees

**Fig. 4:**
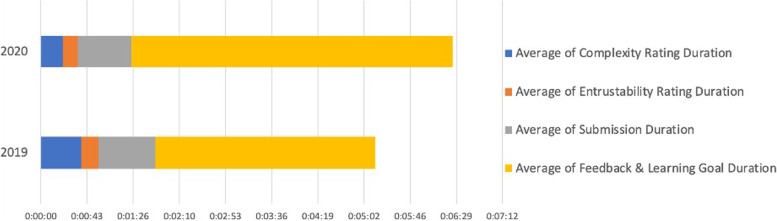
Average duration of ratings for supervisors

The average duration per rating with regard to complexity of the EPA and level of supervision without feedback took 4 minutes and 19 seconds for trainees and 1 minute and 42 seconds for supervisors. The average amount of time per feedback including the definition of new learning goals was 2 minutes and 27 seconds. A complete rating for supervisors thus took 6 minutes and 46 seconds. 93% of all ratings were done completely. These numbers that are illustrated in Figs. 3 and 4 show that the time effort for a rating via the present App is very little. Since it is the trainee who initiates the rating, the time effort for the supervisor is minimal and can be done in less than two minutes if they opt to go without the feedback part. Thus, the App can be applied perfectly in the daily clinical setting. This is proven by the fact that only 9% of all initiated ratings were not finished.

#### Usage of feedback option

**Fig. 5:**
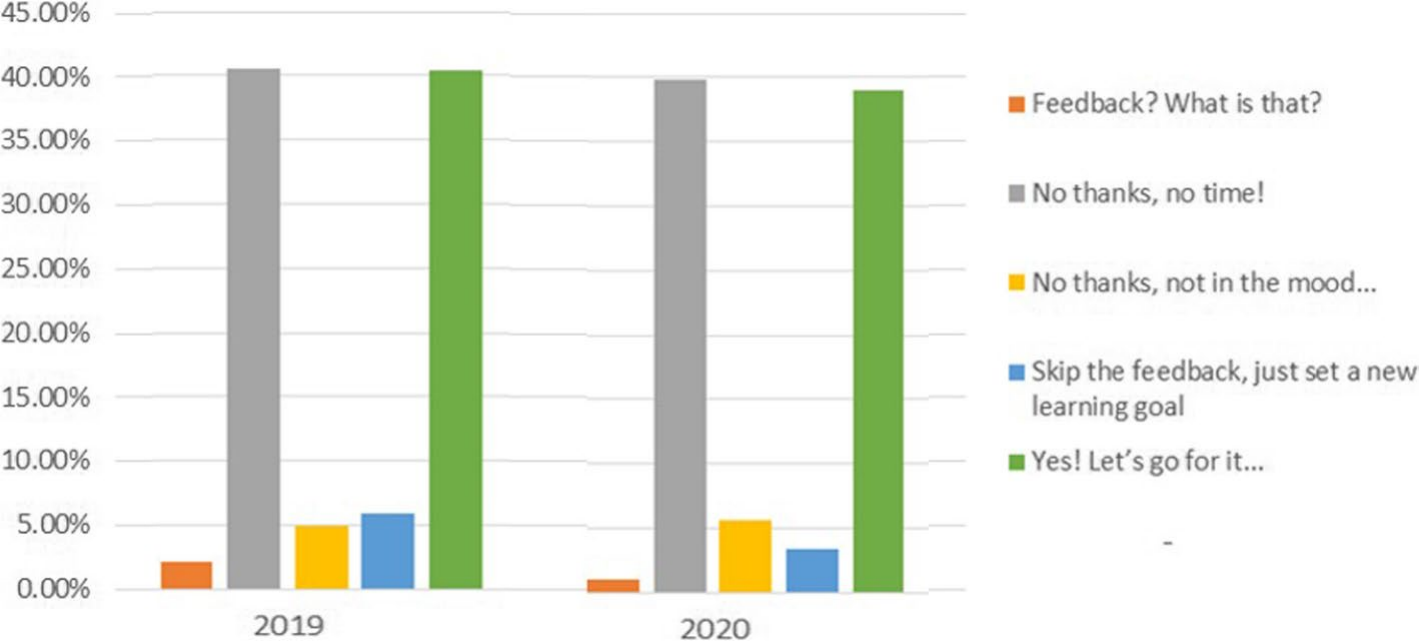
Percentage of feedback options

In 40% of all ratings feedback was given. The answer “No time for feedback” was chosen in 40% of all returned questionnaires. In 6% learning objectives were defined but no feedback was given (see Fig. 5). The surg-prEPAred App is designed for optimizing a conjoint evaluation by trainees and supervisors with respect to the complexity of the EPA and the level of supervision needed. This data by itself is highly valuable for both parties since it shows a clear picture of how one judges the situation. Each data set is a combination of self- and external assessment. The feedback option has been labeled as optional by intent and it is thus highly positive to see that in 50% of all ratings either feedback has been given or learning goals have been defined anyways.

## Discussion and further work

So far, our experience with the App has been positive. After our first go-live, though, a number of improvements were defined to be implemented in future versions of the App. There will be additional levels of supervision in accordance with the ten Cate scale [1]. The App can generally be used in each medical specialty since EPAs can be defined individually according to the specialty’s curriculum requirements. Each trainee can build up a mobile, individual and sustainable competency profile to be used at follow-up training institutions without losing competency when changing positions. Usability will be improved by new graphics for the EPA profile of each trainee, including new legends and management options for learning tools to be defined. In order to generate even more feedback, new feedback options by including pictures or videos are being tested. In future a pdf file of the profile will be generated that can be submitted to educational committees since EPAs are expected to be an integral and mandatory part of medical education. We used the original English version of the application till now, German, French and Italian translations of the interface are just created. Due to the very good first experience with the Surg-prEPAred App in our teaching hospital, the next goal should be to implement both EPAs and App into the specialty training on a national level. The Swiss College of Surgeons intend to implement their new EPA-based core curriculum with the Surg-prEPAred app. Since the use of mobile technology for documenting WBA’s is also part of the newest consensus statements on assessment in medical education [19, 20], several specialties in Switzerland are now using prEPAred to gain experience in working with EPAs. Several studies on this topic are on the way.

## Conclusion

The Surg-prEPAred App is a very valuable, effective and feasible tool to evaluate trainees in a busy clinical setting. The feedback is in “real-time” and thus more specific and meaningful. Thanks to an individual competence portfolio each resident is always aware of her/his level of training and the next steps in surgical training. Supervisors are now capable of customizing their supervision and teaching in accordance with the competence level of the residents.

Through secure transfer and storage of data, the data security of all data can be assured. Thanks to Surg-prEPAred, we are one step closer to “Precision Medical Education”. Our expectation is that the Surg-prEPAred App will eventually also contribute to an improvement of quality of medical education and thus to the quality of patient care and safety.

## Supplementary Information

**Additional file 1.**

## Data Availability

All data used and analyzed during the current study are available from the corresponding author on reasonable request.

## Abbreviations

DOPS: Direct Observation of Procedural Skills
EPAs: Entrustable Professional Activities
GDPR: General Data Protection Regulation
Mini-CEX: Mini-Clinical Evaluation Exercise
SIWF: Swiss Institute for Postgraduate and Further Education in Medicine
WBA: Workplace-based assessment
