# Supplementary material for: Surgery goes EPA (Entrustable Professional Activity) – how a strikingly easy to use app revolutionizes assessments of clinical skills in surgical training
Source: BMC Med Educ. 2022 Jul 19;22:559. doi: 10.1186/s12909-022-03622-1 (PMC9295378; doi:10.1186/s12909-022-03622-1)
Supplement: Supplementary file 1 — Additional file 1. [file 12909_2022_3622_MOESM1_ESM.zip › Supplementary/Post-Survey_Surg-prEPAred_supervisors.pdf]

Surg-prEPAred Post-Survey (supervisors)

Think about the last 4 months:

* 1. Did you use the prEPAred Assessment App at some point during the last 6 months?

- [ ] Yes
- [ ] No

Surg-prEPAred Post-Survey (supervisors)

Evaluating the Usability of the prEPAred-APP

please rate the following statements

2. I would like to use this APP frequently

- ○ strongly agree
- ○ agree
- ○ neutral
- ○ disagree
- ○ strongly disagree

3. I thought the APP was easy to use

- ○ strongly agree
- ○ agree
- ○ neutral
- ○ disagree
- ○ strongly disagree

4. I found the APP to be too complex

- ○ strongly agree
- ○ agree
- ○ neutral
- ○ disagree
- ○ strongly disagree

5. I think that I would need the support of a technical person to be able to use this APP

- ○ strongly agree
- ○ agree
- ○ neutral
- ○ disagree
- ○ strongly disagree

6. I would imagine that most people would learn to use this APP very quickly

- ○ strongly agree
- ○ agree
- ○ neutral
- ○ disagree
- ○ strongly disagree

7. I found the APP very awkward to use

- strongly agree
- agree
- neutral
- disagree
- strongly disagree

8. I felt very confident using the APP

- strongly agree
- agree
- neutral
- disagree
- strongly disagree

9. I needed to spend a lot of time with the APP before I could use it effectively

- strongly agree
- agree
- neutral
- disagree
- strongly disagree

10. I would use this APP frequently to submit short workplace-based assessments

- strongly agree
- agree
- neutral
- disagree
- strongly disagree

11. Assessment data collected from the APP are useful to evaluate the competency of trainees

- strongly agree
- agree
- neutral
- disagree
- strongly disagree

12. I prefer the style of evaluation in the APP compared to the other assessments I am supposed to complete

- strongly agree
- agree
- neutral
- disagree
- strongly disagree

13. It is helpful to see the rating of the trainee next to the one of the supervisor

- strongly agree
- agree
- neutral
- disagree
- strongly disagree

14. I discussed with trainee why I assigned the evaluation score (complexity and "level of supervision")

- all the time
- most of the times
- frequently
- sometimes (infrequently)
- never

15. Overall, I would rate the user-friendliness of this APP as:

- excellent
- good
- neutral
- poor
- awful

16. How many times did you use the APP?

- 0-5
- 6-10
- 11-20
- 21-50
- 51-100
- >100

Surg-prEPAred Post-Survey (supervisors)

* 17. Why didn't you use the APP? (more than one answer is possible)

- I was not interested in this new assessment system
- I was technically not able to use the app (e.g. old phone, no memory)
- I was concerned about the data safety
- other reason (please let us know why. we want to learn!)
- It was too complicated to download and install it
- I was too busy

Surg-prEPAred Post-Survey (supervisors)

Thank you very much for participating!

18. Any other comments and thoughts you want to share about the prEPAred assessment system?
We would appreciate it very much!
