# Supplementary material for: Surgery goes EPA (Entrustable Professional Activity) – how a strikingly easy to use app revolutionizes assessments of clinical skills in surgical training
Source: BMC Med Educ. 2022 Jul 19;22:559. doi: 10.1186/s12909-022-03622-1 (PMC9295378; doi:10.1186/s12909-022-03622-1)
Supplement: Supplementary file 1 — Additional file 1. [file 12909_2022_3622_MOESM1_ESM.zip › Supplementary/Post-Survey_Surg-prEPAred_trainees.pdf]

Surg-prEPAred Post-Survey (trainees)

Think about the last 6 months:
Please rate the following to the best of your ability

(If you are new to your institution, please rate how you usually receive feedback by supervisors)

* 1. The frequency with which I receive feedback from supervisors is:

- never
- rarely
- not frequent enough
- frequent enough
- very frequently

* 2. I am satisfied with the frequency of feedback I receive:

- strongly disagree
- disagree
- neutral
- agree
- strongly agree

* 3. On average, the timeliness of the feedback I receive from faculty is:

- months later
- weeks later
- within a week
- same day
- immediate

* 4. I am satisfied with the timeliness of the feedback I receive:

- strongly disagree
- disagree
- neutral
- agree
- strongly agree

* 5. When I receive feedback, it is limited in amount:

- strongly disagree
- disagree
- neutral
- agree
- strongly agree

* 6. When I receive feedback, it is too general:

- strongly disagree
- disagree
- neutral
- agree
- strongly agree

* 7. When I receive feedback, it does not include an action plan:

- strongly disagree
- disagree
- neutral
- agree
- strongly agree

* 8. When I receive feedback, the supervisors dominate the conversation:

- strongly disagree
- disagree
- neutral
- agree
- strongly agree

* 9. The quality of the feedback I receive from my supervisors is:

- very low quality
- low quality
- moderate quality
- high quality
- very high quality

* 10. I am satisfied with the quality of the feedback I receive:

- strongly disagree
- disagree
- neutral
- agree
- strongly agree

* 11. For how long have you been in postgraduate anesthesiology training?

* 12. For how long have you been working at your current institution?

* 13. Did you use the prEPAred Assessment App at some point during the last 4 months?

- Yes
- No

Surg-prEPAred Post-Survey (trainees)

Evaluating the Usability of the prEPAred-APP

please rate the following statements

14. I would like to use this APP frequently

- strongly agree
- agree
- neutral
- disagree
- strongly disagree

15. I thought the APP was easy to use

- strongly agree
- agree
- neutral
- disagree
- strongly disagree

16. I found the APP to be too complex

- strongly agree
- agree
- neutral
- disagree
- strongly disagree

17. I think that I would need the support of a technical person to be able to use this APP

- strongly agree
- agree
- neutral
- disagree
- strongly disagree

18. I would imagine that most people would learn to use this APP very quickly

- strongly agree
- agree
- neutral
- disagree
- strongly disagree

19. I found the APP very awkward to use

- strongly agree
- agree
- neutral
- disagree
- strongly disagree

20. I felt very confident using the APP

- strongly agree
- agree
- neutral
- disagree
- strongly disagree

21. I needed to spend a lot of time with the APP before I could use it effectively

- strongly agree
- agree
- neutral
- disagree
- strongly disagree

22. I would use this APP frequently to submit short workplace-based assessments

- strongly agree
- agree
- neutral
- disagree
- strongly disagree

23. Assessment data collected from the APP are useful to evaluate the competency of trainees

- strongly agree
- agree
- neutral
- disagree
- strongly disagree

24. I prefer the style of evaluation in the APP compared to the other assessments I am supposed to complete

- ◯ strongly agree
- ◯ agree
- ◯ neutral
- ◯ disagree
- ◯ strongly disagree

25. It is helpful to see the rating of the trainee next to the one of the supervisor

- ◯ strongly agree
- ◯ agree
- ◯ neutral
- ◯ disagree
- ◯ strongly disagree

26. I discussed with supervisor why I assigned the evaluation score (complexity and "level of supervision")

- ◯ all the time
- ◯ most of the times
- ◯ frequently
- ◯ sometimes (infrequently)
- ◯ never

27. Overall, I would rate the user-friendliness of this APP as:

- ◯ excellent
- ◯ good
- ◯ neutral
- ◯ poor
- ◯ awful

28. How many times did you use the APP?

- ◯ 0-5
- ◯ 6-10
- ◯ 11-20
- ◯ 21-50
- ◯ 51-100
- ◯ >100

Surg-prEPAred Post-Survey (trainees)

* 29. Why didn't you use the APP? (more than one answer is possible)

- ☐ I was not interested in this new assessment system
- ☐ I was technically not able to use the app (e.g. old phone, no memory)
- ☐ I was concerned about the data safety
- ☐ other reason (please let us know why. we want to learn!)
- ☐ It was too complicated to download and install it
- ☐ I was too busy

Surg-prEPAred Post-Survey (trainees)

Thank you very much for participating!

30. Any other comments and thoughts you want to share about the prEPAred assessment system?

We would appreciate it very much!
